# Orexin-A controls sympathetic activity and eating behavior

**DOI:** 10.3389/fpsyg.2014.00997

**Published:** 2014-09-08

**Authors:** Giovanni Messina, Carmine Dalia, Domenico Tafuri, Vincenzo Monda, Filomena Palmieri, Amelia Dato, Angelo Russo, Saverio De Blasio, Antonietta Messina, Vincenzo De Luca, Sergio Chieffi, Marcellino Monda

**Affiliations:** ^1^Section of Human Physiology and Clinical Dietetic Service, Department of Experimental Medicine, Second University of NaplesNaples, Italy; ^2^Faculty of Motor Sciences, Parthenope University of NaplesNaples, Italy; ^3^Department of Psychiatry, University of TorontoToronto, ON, Canada

**Keywords:** body weight, orexin-A, energy expenditure, sympathetic nervous system, behavior

## Abstract

It is extremely important for the health to understand the regulatory mechanisms of energy expenditure. These regulatory mechanisms play a central role in the pathogenesis of body weight alteration. The hypothalamus integrates nutritional information derived from all peripheral organs. This region of the brain controls hormonal secretions and neural pathways of the brainstem. Orexin-A is a hypothalamic neuropeptide involved in the regulation of feeding behavior, sleep-wakefulness rhythm, and neuroendocrine homeostasis. This neuropeptide is involved in the control of the sympathetic activation, blood pressure, metabolic status, and blood glucose level. This minireview focuses on relationship between the sympathetic nervous system and orexin-A in the control of eating behavior and energy expenditure. The “thermoregulatory hypothesis” of food intake is analyzed, underlining the role played by orexin-A in the control of food intake related to body temperature. Furthermore, the paradoxical eating behavior induced orexin-A is illustrated in this minireview.

## INTRODUCTION

Obesity and diabetes are a worldwide public health issue with extensive medical, social, and economic consequences (Yach et al., 2006; Runge, 2007). Obesity (body mass index ≥30 kg of body weight/m^2^ of height) has negative effects on health and increases the risk of developing a variety of diseases, including cardiovascular syndromes, some cancers, and diabetes mellitus (Must et al., 1999; Field et al., 2001; Calle et al., 2003; Friedenberg et al., 2008). Over the past three decades, the prevalence of obesity has doubled in the USA and in Europe (Ogden et al., 2006; Van Vliet-Ostaptchouk et al., 2014). Although according to the most recent data published in the 2005–2006 update of the National Health and Nutrition Examination Survey (NHANES) obesity rates have stabilized, others (Wang and Beydoun, 2007) expect that the obesity “epidemic” will only continue to worsen, with as many as 75% of Americans and of Europeans potentially being overweight in the year 2020. Physicians will undoubtedly encounter obese people in clinical practice and must, then, be able to identify and address care needs specific to this patient population.

This minireview focuses on relationship between the autonomic nervous system and orexin-A in the control of eating behavior, energy expenditure, and body weight regulation. The “thermoregulatory hypothesis” of food intake (Himms-Hagen, 1995) is analyzed, underlining the role played by orexin-A in the control of eating behavior related to body temperature.

## ENERGY HOMEOSTASIS

Energy homeostasis is determined by the balance between intake of calories and energy expenditure. This is regulated by interconnected neuroendocrine and autonomic pathways (Monda et al., 2008a).

Resting energy expenditure (REE) accounts for 60–75% of total daily energy expenditure. Several factors contribute to the inter-individual variability in REE such as fat-free mass (FFM; Weyer et al., 1999), sympathetic nervous system (SNS) activity (Welle et al., 1991; Messina et al., 2012), and endocrine status [e.g., thyroid hormone (Danforth and Burger, 1984)]. REE decreases with age (Roubenoff et al., 2000). This decline is due not only to the loss of FFM and an alteration in its metabolically active components, but also to the reduction in physical activity.

## HYPOTHALAMUS AND OREXINS

The hypothalamus, a key component for regulation of energy homeostasis, continuously monitors signals that reflect energy status and initiates appropriate behavioral and metabolic responses (Suzuki et al., 2012). It controls glucose utilization in insulin-sensitive organs, such as skeletal muscle, as well as whole-body energy metabolism (Sudo et al., 1991; Haque et al., 1999).

**FIGURE 1:**
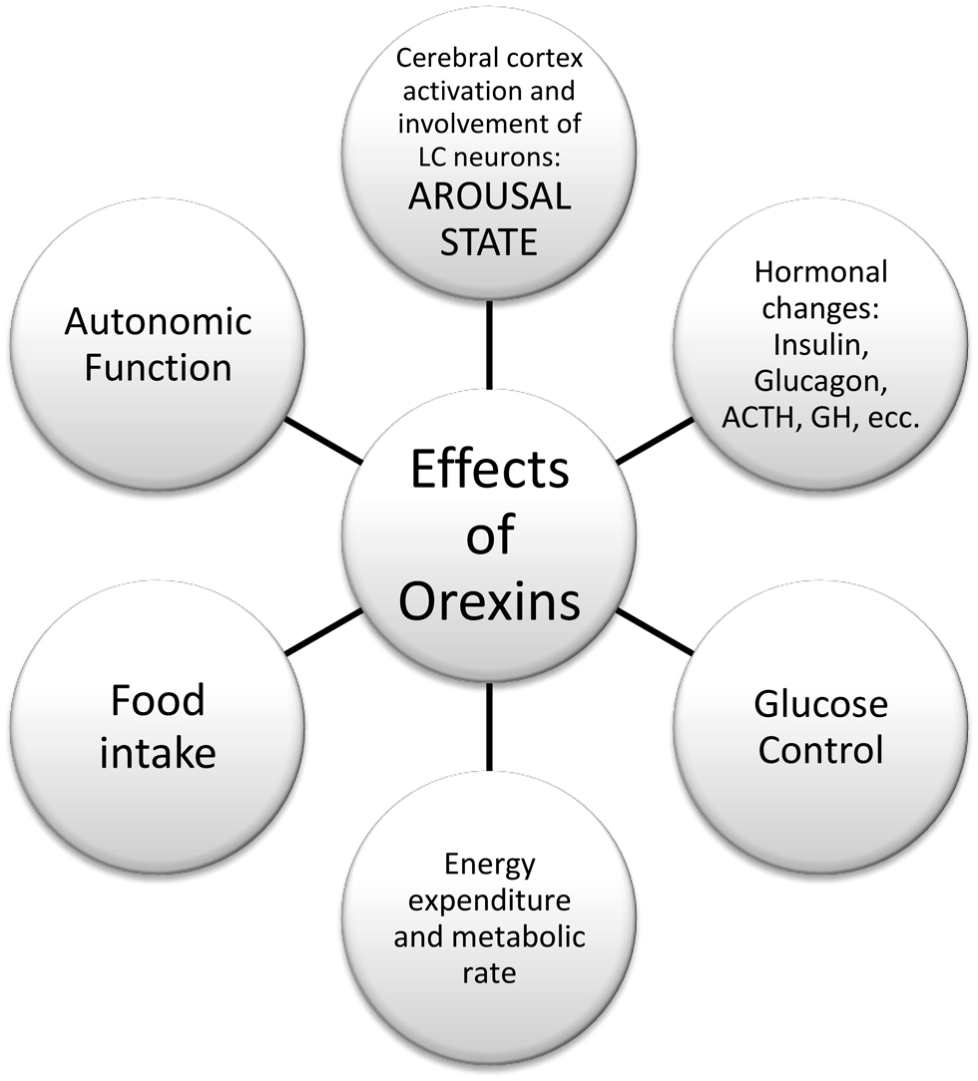
**Effects of orexins in peripheral tissue and central nervous system**.

Orexins A and B are hypothalamic neuropeptides, involved in the regulation of feeding behavior, sleep-wakefulness rhythm, and neuroendocrine homeostasis (Kukkonen et al., 2002; Monda et al., 2005; Viggiano et al., 2006), as reported in **Figure 1**.

These peptides derive from the prepro-orexin (preprohypocretin) gene, which encodes a precursor (130 amino acids in rodents, 131 residues in humans) that is cleaved into orexin-A (synonymous with hypocretin-1; 33 amino acids) and orexin-B (hypocretin-2; 28 residues; Sakurai et al., 1998). Orexins promote both arousal and feeding (Sweet et al., 1999). Orexin-A binds to two G-protein-coupled receptors, orexin receptor-1 (hypocretin receptor-1) and orexin receptor-2 (hypocretin receptor-2). The expression pattern of mRNA encoding two orexin receptors (OX1R andOX2R) in the rat’s brain has been demonstrated (Trivedi et al., 1998; Machaalani et al., 2013). Within the hypothalamus, expression for the OX1R mRNA was largely restricted in the ventromedial (VMH) and dorsomedial hypothalamic nuclei, while paraventricular nucleus, VMH, and arcuate nucleus contain high levels of OX2R mRNA, as well as in mammillary nuclei (Zhang et al., 2005). Lu et al. (2000) have demonstrated that levels of OX1R mRNA significantly increased in the VMH of rats after 20 h of fasting. An initial decrease (14 h) and a subsequent increase (20 h) in OX1R mRNA levels after fasting were observed in the dorsomedial hypothalamic nucleus. Levels of OX2R mRNA increased in the arcuate nucleus, but they didn’t change in the dorsomedial hypothalamic nucleus and paraventricular hypothalamic nucleus following fasting (Lu et al., 2000).

Orexin neurons may also functionally interact with glucose-sensitive neurons in the hypothalamus, notably the glucose-responsive cells (glucose-excited neurons: stimulated by rising glucose levels) found predominantly in the VMH, and the glucose-sensitive neurons (glucose-inhibited neurons: stimulated when glucose falls) that constitute 30% of lateral hypothalamic area (LHA) neurons. There are synaptic contacts between orexin neurons and glucose-sensitive cells in the LHA (Shiraishi et al., 2000), while orexin-A specifically stimulates the glucose-sensitive cells (Liu et al., 2001). On the contrary, orexin-A inhibits glucose-responsive neurons in the VMH (Shiraishi et al., 2000). Muroya et al. (2001) suggest that some glucose-sensitive neurons express orexins. In the medulla, orexin neurons innervate not only the ventral area (Zheng et al., 2005), but also the nucleus of the solitary tract (Ciriello et al., 2003), which is an important relay station that receives sensory signals, such as portal vein glucose availability and gastric distension from the viscera. These signals are conveyed to the hypothalamus (Horst et al., 1989).

Sugar-sensing neurons exist in restricted brain regions, such as hypothalamus and brain stem, and they are classified into two groups, called glucose-excited (GE) neurons and glucose-inhibited (GI) neurons, in terms of the mode of response to extracellular glucose changes within physiological cerebrospinal fluid (CSF) range (Burdakov and González, 2009; Gonzàlez et al., 2009). For instance, orexin neurons in the LHA and neuropeptide Y (NPY)/agouti-related peptide (AgRP) neurons in the ARC are glucose-inhibited, whereas melanin-concentrating hormone (MCH) neurons in LHA and proopiomelanocortin (POMC) neurons in the ARC are glucose-excited (Burdakov et al., 2005; Burdakov and González, 2009). The sugar sensing of orexin neurons, which is a major class of GI neurons, is metabolism-independent, since the glucose response is unaffected by glucokinase inhibitors, and mimicked by a non-metabolizable glucose analog 2-deoxyglucose (González et al., 2008), although the accurate mechanisms, particularly the functional molecules relevant to glucose-induced inhibition, have not yet been explained. Orexin neurons are not inhibited by L-glucose, galactose, α-methyl-D-glucoside, or fructose, whereas GE neurons can sense galactose. More recently, it has been suggested that orexin neurons function as a “conditional glucosensor,” because the electrical activity of orexin neurons is more potently inhibited by glucose when intracellular energy levels (i.e., cytosolic levels of pyruvate, lactate, or ATP) are low, whereas high energy levels attenuate the glucose response in orexin neurons (Venner et al., 2011). Besides, Yi et al. (2009) have reported that a continuous intracerebroventricula (ICV) infusion of orexin-A (1 mmol/L, 5 μL/h) into rats fasted for 5 h brought about an increase in plasma glucose levels, and prevented a daytime decrease of endogenous hepatic glucose production (EGP). Hepatic sympathetic, but not parasympathetic, denervation blocked the orexin induced apparent enhancement of EGP.

In addition, when the γ-aminobutyric acid receptor antagonist bicuculline was administered in the perifornical area in order to activate orexin neurons, basal EGP was increased, and insulin-mediated suppression of EGP was attenuated, but the insulin-induced glucose disposal was enhanced (Yi et al., 2009).

In addition, the presence of orexin receptors in other cerebral areas suggests that orexin-A plays additional functions (Kukkonen et al., 2002). It has been demonstrated that the orexins play a role in sleep regulation (Beuckmann and Yanagisawa, 2002). Deficiency in orexin neurotransmission results in the sleep disorder narcolepsy in mice, dogs, and humans (Monda et al., 2004a). Orexin derangements in patients with narcolepsy were associated with an increased body mass index (Schuld et al., 2000) and a higher risk of type-II diabetes mellitus (Honda et al., 1996).

Orexins exert peripheral effect and this was suggested by the detection of substantial levels of orexins in plasma (Adam et al., 2002), as well as the presence of orexin receptors in several peripheral tissues, including the gastrointestinal tract (GIT), endocrine pancreas, adrenal glands, and adipose tissue (Digby et al., 2006; Heinonen et al., 2008).

Snow et al. (2002) have demonstrated that plasma orexin levels are one-fifth to one-eighth of orexin CSF values. However, the source of orexin in peripheral tissue is still unclear. Is orexin directly released into the blood stream or leaked from the CSF? One possibility is that orexin is released from the brain. The other possibility is that orexin is produced directly in peripheral tissues. Orexin-immunoreactive cells are observed in the gastrointestinal tract and pancreas. However, the question of orexin synthesis in peripheral tissue is still under discussion. Further studies are needed to better understand orexin physiology in peripheral tissues.

The influence of orexin-A on metabolic status and plasma glucose level may contribute to increase diabetics morbidity and mortality (Minokoshi et al., 1999). It has been proved that orexins affect the plasma lipoprotein profile and insulin glucose homeostasis (Muroya et al., 2001). Orexins stimulate insulin release from pancreatic cells *in vivo* and *in vitro* (Nowak et al., 2000). Several studies have focused on finding out the relationship between circulating orexin and fat mass and have proved that there is a strong correlation between low plasma orexin and obesity (Adam et al., 2002; Messina et al., 2013a). A significant issue is whether this naturally occurring biological peptide “orexin” in useful in weight management or obesity treatment. Many suggest that when orexin is peripherally injected, it activates thermogenesis, without limiting feeding or increasing physical activity. These encouraging observations have paved the way for clinical testing of the thermogenic potential of orexin (Messina et al., 2013b).

Orexin-A controls glucose production and utilization in the peripheral tissues via the autonomic nervous system (Tsuneki et al., 2010). These conclusions demonstrate that orexin is involved in the control of central and peripheral hormonal actions for the maintenance of glucose homeostasis, though it has been demonstrated that glucose control remains following decerebration (DiRocco and Grill, 1979). Existing evidence suggests that orexins induce glucose production in the liver (Stanley et al., 2010) and help glucose uptake in skeletal muscle (Yi et al., 2009). In addition it has been shown that orexins A and B differentially regulate glucagon release from pancreas (Bass and Takahashi, 2010).

**FIGURE 2:**
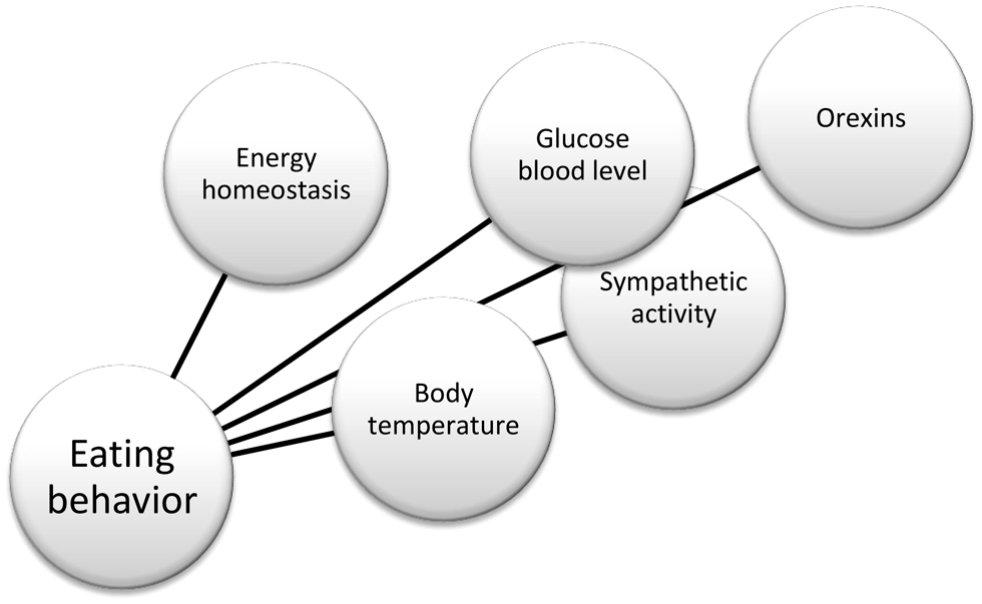
**Factors influencing eating behavior**.

In summary, there is substantial evidence in the literature that helps to define the physiological role of orexin neurons, and their connections, as reported in **Figure 2**. For instance, anatomical works by Kilduff and Peyron (2000) and Kerman (2008); physiological studies by Karnani and Burdakov (2011) and Inutsuka and Yamanaka (2013) in glucose-regulation, and Morrison et al. (2012a) in thermoregulation. More recently, opto- and pharmaco-genetic tools also have been used to investigate the physiological role of these neurons (Heydendael et al., 2014; Inutsuka et al., 2014). Finally, hypothalamic orexin neurons co-express glutamate vesicular transporters (Rosin et al., 2003), suggesting an important role of this neurotransmitter in the orexinergic pathway.

## THE SYMPATHETIC NERVOUS SYSTEM

Eating is a complex behavior that partly involves the sympathetic nervous system. This sympathetic involvement is exerted by an influence on body temperature, in agreement with the “thermoregulatory hypothesis” of eating behavior (Himms-Hagen, 1995). Obviously, the role of the sympathetic system in controlling the eating behavior is not restricted only to changes in body temperature. For instance, the aforementioned glucose-control involves the sympathetic system.

Orexin-A also influences body temperature. In fact, an ICV administration of orexin-A induces an increase in firing rate of the sympathetic nerves to BAT, accompanied with a rise in BAT and colonic temperatures (Monda et al., 2001). The simultaneous increase in heart rate and body temperature after an ICV injection of orexin-A shows a generalized activation of the sympathetic nervous system. Few studies have been made on the topic of the roles played by different cerebral areas involved in the induction of the above-mentioned tachycardia and hyperthermia (Monda et al., 1994, 1995, 1996).

The sympathetic adjustment of thermoregulation also implies in energy expenditure. The functional organization and neurochemical influences within the CNS networks governs the level of BAT sympathetic nerve activity to produce the thermoregulatory and metabolically driven alterations in BAT thermogenesis and energy expenditure that contribute to overall energy homeostasis (Morrison et al., 2014). BAT thermogenesis contributes to the maintenance of body temperature during cold exposure and to the elevated core temperature during several behavioral states, including wakefulness, the acute phase response (fever), and stress. BAT energy expenditure requires metabolic fuel availability and contributes to energy balance.

The consequences of the “thermoregulatory hypothesis” of eating behavior are that subjects with a high set-point of body temperature and/or low sympathetic activity are induced to eat a high quantity of food to elevate the sympathetic discharge and body temperature. Many studies (Keesey and Hirvonen, 1997; Morrison et al., 2012b) indicate that some forms of obesity can be regarded as instances of regulation at an elevated set point, while other forms seemingly result from a regulatory dysfunction, as already reported by Keesey (1988).

Conversely, subjects with a low thermal set-point and/or a high sympathetic tone need to introduce a lower quantity of food to reach a prefixed thermal set-point. Alterations of postprandial thermogenesis due to a reduced response of sympathetic activation can play an important role in inducing obesity. In other words, subjects with a low postprandial sympathetic activation need to introduce a higher quantity of foot to reach a prefixed body temperature. On the other hand, being overweight increases the sympathetic discharge that contributes to induce diseases related to abnormal body weight (Lambert et al., 2010).

Chronic sympathetic over activity is also known to be present in central obesity, and many evidences demonstrate the consequence of a high sympathetic outflow to organs such as the heart, kidneys, and blood vessels. Chronic sympathetic nervous system over activity can also contribute to a further decline of insulin sensitivity, creating a vicious cycle that may lead to the development of the metabolic syndrome and hypertension. The cause of this over activity is not clear, but may be driven by certain adipokines (Smith and Minson, 2012). In addition, the postprandial activation of the peripheral sympathetic nervous system is fundamental to maintain energy balance. A contribution of postprandial sympathetic activation to the thermic effect of food is not always evident and depends on the size and composition of the meal, with carbohydrates having the clearest effect. Signals related to food intake from various origins (e.g., gut, hepatoportal area, chemoreceptors) are integrated in the brain and result in increased peripheral sympathetic outflow. It is of interest to emphasize the role of diet composition (according to the life style of subjects) in the level of sympathetic activation during the day in view of the potential role of adrenergic over activity in the pathogenesis of obesity and its metabolic syndrome (Van Baak, 2008).

Power spectral analysis (PSA) of the heart rate variability (HRV) is considered a non-invasive method for quantitative and qualitative evaluation of the autonomic nervous system activity in various fields of research and clinical studies. In the frequency domain method of HRV, the high frequency (HF) component is associated solely with parasympathetic activity. The low frequency (LF) component is associated with both sympathetic and parasympathetic activities, but sympathetic activity is the greater contributor. LF power may correlate more with baroreflex function and/or stress that with the cardiac sympathetic innervations (Moak et al., 2007; Shah et al., 2011).

This approach should modify the interpretations about the sympathetic function in the pathophysiology of the obesity. In a study conducted in our laboratory (Monda et al., 2006a), we demonstrated that LF and HF values of premenopausal obese women were lower than values of lean women. In postmenopause, LF and HF have a comparable decline in lean and obese women, as a consequence no difference can be found. These results suggest a reduction of the vegetative modulation in obese young women and the reduction of the autonomic control regards both the sympathetic and parasympathetic components (Monda et al., 2006b). The reduction of the sympathetic branch could be an important factor in the maintenance of obesity in premenopausal age. Indeed, a reduction in the sympathetic activity could be linked to a low energy expenditure, so that a reduced energetic cost could explain the higher body weight in premenopausal women. In this experiment, the autonomic activity of postmenopausal women is lower than that of premenopausal subjects, though a better indicator of the sympathetic activity would be very low frequencies (Fleisher et al., 1996). This indicates that the modifications of the autonomic modulation cannot be included among factors related to obesity in postmenopausal subjects. Many experimental evidences have demonstrated that an increase in sympathetic and thermogenic activity reduces food intake. Therefore, the obesity can be due to an increase in food intake associated to a reduced activity of the sympathetic nervous system. On the other hand, a study revealed lower respiratory sinus arrhythmia, as evaluated by the HF-HRV spectral analysis combined with deep breathing tests, which points to the presence of cardiac vagal dysfunction in obese adolescents (Tonhajzerova et al., 2008). Importantly, autonomic imbalance with decreased parasympathetic activity maybe the final common pathway in numerous conditions associated with increased morbidity and mortality (Thayer and Lane, 2007). The evaluation of cardio respiratory interactions, in particular the heart rate variability, can provide diagnostic information about early subclinical autonomic dysfunction in obesity. Traditionally, there have been two hypotheses about the nature of the predominate abnormality in SNS behavior in human obesity. Bray (1991) used the acronym “MONA LISA” to describe his hypothesis that Most Obesities kNown Are Low In Sympathetic Activity. This vision was based principally on studies in rodents that exhibited low SNS activity and morbid obesity following lesions in the ventromedial hypothalamus. As such, low SNS activity was considered causal in the development of obesity. In contrast, Landsberg (1986) viewed SNS activation targeting the heart, blood vessels and kidneys as a critical relation to the well documented relation between obesity and hypertension (Hall, 2003; Davy and Hall, 2004).

## PARADOXICAL EATING BEHAVIOR: HYPERPHAGIA AND HYPOPHAGIA BY OREXIN-A

Since orexin-A is able to induce both the activation of thermogenesis and hyperphagia, Monda et al. (2003) tested the possibility that a previous thermogenic activation induced by orexin-A can modify eating behavior. Food intake and body temperature were monitored in 24 h-fasting male Sprague-Dawley rats for 15 h after food presentation during the dark period. Orexin-A was injected into the lateral cerebral ventricle 6 h before food presentation. Food intake and body temperature were controlled also in rats receiving orexin-A at the same time of food presentation. Orexin-A caused the same elevation of body temperature in both groups, while food intake was significantly lower in the group receiving orexin-A 6 h before food presentation in comparison to the other group. This study demonstrated that the effects on food intake induced by orexin-A depend on the time of food presentation. This suggest to revise the role of orexin-A in the control of food intake. The name assigned to this peptide was due to the strong increase in food intake after an orexin-A administration, assigning a fundamental role in the induction of food intake (Wolf, 1998; Shiraishi et al., 2000). The results of the above publication call for a re-discussion of this role, underlining the importance of orexin-A in the control of the sympathetic activity and body temperature, which in turn affects food intake. An ICV injection of orexin-A induces an increase in the sympathetic activity and in the body temperature independently of food ingestion, that is reduced in the rats with a delayed presentation of food. This suggests that the effects on body temperature are prevalent with respect to eating behavior. Then, orexin-A can induce hyperphagia, but also hypophagia, contradicting the significance of this name that assign a primary hyperphagic effect to this peptide. For this reason, orexin-A cannot be considered a substance with a primary hyperphagic effect.

Orexin-A can induce hypophagia or hyperphagia (Shiraishi et al., 2000), but it always induces an activation of thermogenesis (Monda et al., 2004b, 2008b). We believe that this peptide elevates the thermoregulatory set-point, inducing the reactions to reach the new level of body temperature. The increase in food intake, obtained in the rats with a non-delayed presentation of food, could be a reaction aimed to reach an elevated body temperature. Indeed, food ingestion induces a rise in body temperature due to postprandial thermogenesis (De Luca et al., 1987; Tentolouris et al., 2006; Monda et al., 2008a; Messina et al., 2012, 2013a). The hyperphagic effect of orexin-A disappears when the body temperature is already increased, so that a reduction in food intake can happen in this condition.

Although selective activation of orexin neurons directly can elicit eating behavior (Inutsuka et al., 2014), the above-reported demonstrations support the idea that orexin-A controls body temperature and subsequently eating behavior.

## Conflict of Interest Statement

The authors declare that the research was conducted in the absence of any commercial or financial relationships that could be construed as a potential conflict of interest.

## References

[B1] Adam J. A. Menheere P. P. C. A. Van Dielen F. M. H. Soeters P. B. Buurman W. A. Greve G. W. (2002). Decreased plasma orexin-A levels in obese individuals. *Int. J. Obes.* 26 274–266 10.1038/sj.ijo.0801868 11850761

[B2] Bass J. Takahashi J. S. (2010). Circadian integration of metabolism and energetics. *Science* 330 1349–1354 10.1126/science.1195027 21127246 PMC3756146

[B3] Beuckmann C. T. Yanagisawa M. (2002). Orexins: from neuropeptides to energy homeostasis and sleep/wake regulation. *J. Mol. Med.* 80 329–342 10.1007/s00109-002-0322-x 12072908

[B4] Bray G. A. (1991). Obesity, a disorder of nutrient partitioning: the MONA LISA hypothesis. *J. Nutr.* 121 1146–1162 1861165 10.1093/jn/121.8.1146

[B5] Burdakov D. González J. A. (2009). Physiological functions of glucose-inhibited neurones. *Acta Physiol. (Oxf.)* 195 71–78 10.1111/j.1748-1716.2008.01922.x 18983451 PMC5767113

[B6] Burdakov D. Luckman S. M. Verkhratsky A. (2005). Glucose-sensing neurons of the hypothalamus. *Philos. Trans. R. Soc. Lond. B Biol. Sci.* 360 2227–2235 10.1098/rstb.2005.1763 16321792 PMC1569598

[B7] Calle E. E. Rodriguez C. Walker-Thurmond K. Thun M. J. (2003). Overweight, obesity, and mortality from cancer in a prospectively studied cohort of U.S. adults. *N. Engl. J. Med.* 348 1625–1638 10.1056/NEJMoa021423 12711737

[B8] Ciriello J. McMurray J. C. Babic T. de Oliveira C. V. (2003). Collateral axonal projections from hypothalamic hypocretin neurons to cardiovascular sites in nucleus ambiguus and nucleus tractus solitarius. *Brain Res.* 991 133–141 10.1016/j.brainres.2003.08.016 14575885

[B9] Danforth E. Jr. Burger A. (1984). The role of thyroid hormones in the control of energy expenditure. *Clin. Endocrinol. Metab.* 13 581–595 10.1016/S0300-595X(84)80039-0 6391756

[B10] Davy K. P. Hall J. E. (2004). Obesity and hypertension: two epidemics or one? *Am.* *J. Physiol. Regul. Integr. Comp. Physiol.* 286 R803–R813 10.1152/ajpregu.00707.2003 15068965

[B11] De Luca B. Monda M. Pellicano M. P. Zenga A. (1987). Cortical control of thermogenesis induced by lateral hypothalamic lesion and overeating. *Am. J. Physiol. Regul. Integr. Comp. Physiol.* 253 R626–R633 10.1152/ajpregu.1987.253.4.R626 2889375

[B12] Digby J. E. Chen J. Tang J. Y. Lehnert H. Matthews R. N. Randeva H. S. (2006). Orexin receptor expression in human adipose tissue: effects of orexin-A and orexin-B. *J. Endocrinol.* 19 129–136 10.1677/joe.1.06886 17065396

[B13] DiRocco R. J. Grill H. J. (1979). The forebrain is not essential for sympathoadrenal hyperglycemic response to glucoprivation. *Science* 204 1112–1114 10.1126/science.451558 451558

[B14] Field A. E. Coakley E. H. Must A. Spadano J. L. Laird N. Dietz W. H. (2001). Impact of overweight on the risk of developing common chronic diseases during a 10-year period. *Arch. Intern. Med.* 161 1581–1586 10.1001/archinte.161.13.1581 11434789

[B15] Fleisher L. A. Frank S. M. Sessler D. I. Cheng C. Matsukawa T. Vannier C. A. (1996). Thermoregulation and heart rate variability. *Clin. Sci. (Lond.)* 90 97–103 8829887 10.1042/cs0900097

[B16] Friedenberg F. K. Xanthopoulos M. Foster G. D. Richter J. E. (2008). The association between gastroesophageal reflux disease and obesity. *Am. J. Gastroenterol.* 103 2111–2122 10.1111/j.1572-0241.2008.01946.x 18796104

[B17] González J. A. Jensen L. T. Fugger L. Burdakov D. (2008). Metabolism-independent sugar sensing in central orexin neurons. *Diabetes* 57 2569–2576 10.2337/db08-0548 18591392 PMC2551664

[B18] Gonzàlez J. A. Reimann F. Burdakov D. (2009). Dissociation between sensing and metabolism of glucose in sugar sensing neurones. *J. Physiol. (Lond.)* 587 41–48 10.1113/jphysiol.2008.163410 18981030 PMC2670021

[B19] Hall J. E. (2003). The kidney, hypertension, and obesity. *Hypertension* 41 625–633 10.1161/01.HYP.0000052314.95497.78 12623970

[B20] Haque M. S. Minokoshi Y. Hamai M. Iwai M. Horiuchi M. Shimazu T. (1999). Role of the sympathetic nervous system and insulin in enhancing glucose uptake in peripheral tissues after intrahypothalamic injection of leptin in rats. *Diabetes* 48 1706–1712 10.2337/diabetes.48.9.1706 10480598

[B21] Heinonen M. V. Purhonen A. K. Makela A. K. Herzig K. H. (2008). Functions of orexins in peripheral tissues. *Acta Physiol.* 192 471–485 10.1111/j.1748-1716.2008.01836.x 18294339

[B22] Heydendael W. Sengupta A. Beck S. Bhatnagar S. (2014). Optogenetic examination identifies a context-specific role for orexins/hypocretins in anxiety-related behavior. *Physiol. Behav.* 130 182–190 10.1016/j.physbeh.2013.10.005 24140988 PMC4155939

[B23] Himms-Hagen J. (1995). Role of brown adipose tissue thermogenesis in control of thermoregulatory feeding in rats: a new hypothesis that links thermostatic and glucostatic hypotheses for control of food intake. *Proc. Soc. Exp. Biol. Med.* 208 159–169 10.3181/00379727-208-43847A 7831348

[B24] Honda Y. Doi Y. Ninomiya R. Ninomiya C. (1996). Increased frequency of non-insulin-dependent diabetes mellitus among narcoleptic patients. *Sleep* 9 254–259 3518018 10.1093/sleep/9.1.254

[B25] Horst G. J. T. de Boer P. Luiten P. G. M. van Willigen J. D. (1989). Ascending projections from the solitary tract nucleus to the hypothalamus: a phaseolus valgaris lectin tracing study in the rat. *Neuroscience* 31 785–797 10.1016/0306-4522(89)90441-7 2594200

[B26] Inutsuka A. Inui A. Tabuchi S. Tsunematsu T. Lazarus M. Yamanaka A. (2014). Concurrent and robust regulation of feeding behaviors and metabolism by orexin neurons. *Neuropharmacology* 85 451–460 10.1016/j.neuropharm.2014.06.015 24951857

[B27] Inutsuka A. Yamanaka A. (2013). The regulation of sleep and wakefulness by the hypothalamic neuropeptide orexin/hypocretin. *Nagoya J. Med. Sci.* 75 29–36 23544265 PMC4345701

[B28] Karnani M. Burdakov D. (2011). Multiple hypothalamic circuits sense and regulate glucose levels. *Am. J. Physiol.* 300 R47–R55 10.1152/ajpregu.00527.2010 PMC3023280 21048078

[B29] Keesey R. E. (1988). The body-weight set point. What can you tell your patients? *Postgrad. Med.* 83 114–118 3283713 10.1080/00325481.1988.11700259

[B30] Keesey R. E. Hirvonen M. D. (1997). Body weight set-points: determination and adjustment. *J. Nutr.* 127 1875S–1883S 9278574 10.1093/jn/127.9.1875S

[B31] Kerman I. A. (2008). Organization of brain somatomotor-sympathetic circuits. *Exp. Brain Res.* 187 1–16 10.1007/s00221-008-1337-5 18369609

[B32] Kilduff T. S. Peyron C. (2000). The hypocretin/orexin ligand-receptor system: implications for sleep and sleep disorders. *Trends Neurosci.* 23 359–365 10.1016/S0166-2236(00)01594-0 10906799

[B33] Kukkonen J. P. Holmqvist T. Ammoun S. Akerman K. E. (2002). Functions of the orexinergic/hypocretinergic system. *Am. J. Physiol.* 283 1567–1591 10.1152/ajpcell.00055.2002 12419707

[B34] Lambert G. W. Straznicky N. E. Lambert E. A. Dixon J. B. Schlaich M. P. (2010). Sympathetic nervous activation in obesity and the metabolic syndrome-causes, consequences and therapeutic implications. *Pharmacol. Ther.* 126 159–172 10.1016/j.pharmthera.2010.02.002 20171982

[B35] Landsberg L. (1986). Diet, obesity and hypertension: an hypothesis involving insulin, the sympathetic nervous system, and adaptive thermogenesis. *Q. J. Med.* 61 1081–1090 3310065

[B36] Liu X. H. Morris R. Spiller D. White M. Williams G. (2001). Orexin-A preferentially excites glucose-sensitive neurons in the lateral hypothalamus of rats in vitro. *Diabetes* 50 2431–2437 10.2337/diabetes.50.11.2431 11679418

[B37] Lu X. Y. Bagnol D. Burke S. Akil H. Watson S. J. (2000). Differential distribution and regulation of OX1 and OX2 orexin/hypocretin receptor messenger RNA in the brain upon fasting. *Horm. Behav.* 37 335–344 10.1006/hbeh.2000.1584 10860677

[B38] Machaalani R. Hunt N. J. Waters K. A. (2013). Effects of changes in energy homeostasis and exposure of noxious insults on the expression of orexin(hypocretin) and its receptors in the brain. *Brain Res.* 1526 102–122 10.1016/j.brainres.2013.06.035 23830852

[B39] Messina G. De Luca V. Viggiano A. Ascione A. Iannaccone T. Chieffi S. (2013a). Autonomic nervous system in the control of energy balance and body weight: personal contributions. *Neurol. Res. Intern.* 3:639280 10.1155/2013/639280 PMC3649682 23691314

[B40] Messina G. Viggiano A. De Luca V. Messina A. Chieffi S. Monda M. (2013b). Hormonal changes in menopause and orexin-a action. *Obst. Gynec. Intern.* 2013:209812 10.1155/2013/209812 PMC3693173 23840215

[B41] Messina G. Vicidomini C. Viggiano A. Tafuri D. Cozza V. Cibelli G. (2012). Enhanced parasympathetic activity of sportive women is paradoxically associated to enhanced resting energy expenditure. *Auton. Neurosci.* 169 102–106 10.1016/j.autneu.2012.05.003 22682704

[B42] Minokoshi Y. Haque M. S. Shimazu T. (1999). Microinjection of leptin into the ventromedial hypothalamus increases glucose uptake in peripheral tissues in rats. *Diabetes* 48:287291 10.2337/diabetes.48.2.287 10334303

[B43] Moak J. P. Goldstein D. S. Eldadah B. A. Saleem A. Holmes C. Pechnik S. (2007). Supine low-frequency power of heart rate variability reflects baroreflex function, not cardiac sympathetic innervations. *Heart Rhythm* 4 1523–1529 10.1016/j.hrthm.2007.07.019 17997358 PMC2204059

[B44] Monda M. Amaro S. Sullo A. De Luca B. (1994). Posterior hypothalamic activity and cortical control during the PGE1 hyperthermia. *Neuroreport* 6 135–139 10.1097/00001756-199412300-00035 7703402

[B45] Monda M. Amaro S. Sullo A. De Luca B. (1995). Injection of muscimol in the posterior hypothalamus reduces the PGE1-hyperthermia in the rat. *Brain Res. Bull.* 37 575–580 10.1016/0361-9230(95)00032-A 7670880

[B46] Monda M. Messina G. Vicidomini C. Viggiano A. Mangoni C. De Luca B. (2006a). Activity of autonomic nervous system is related to body weight in pre-menopausal, but not in post-menopausal women. *Nutr. Neurosci.* 9 141–145 10.1080/10284150600903552 17176636

[B47] Monda M. Viggiano A. Viggiano A. Viggiano E. Messina G. Tafuri D. (2006b). Quetiapine lowers sympathetic and hyperthermic reactions due to cerebral injection of orexin A. *Neuropeptides* 40 357–363 10.1016/j.npep.2006.07.003 17010428

[B48] Monda M. Messina G. Mangoni C. De Luca B. (2008a). Resting energy expenditure and fat-free mass do not decline during aging in severely obese women. *Clin. Nutr.* 27 657–659 10.1016/j.clnu.2008.04.005 18514973

[B49] Monda M. Viggiano A. Viggiano A. Mondola R. Viggiano E. Messina G. (2008b). Olanzapine blocks the sympathetic and hyperthermic reactions due to cerebral injection of orexin A. *Peptides* 29 120–126 10.1016/j.peptides.2007.10.016 18053616

[B50] Monda M. Sullo A. De Luca E. Pellicano M. P. (1996). Lysine acetylsalicylate modifies aphagia and thermogenic changes induced by lateral hypothalamic lesion. *Am. J. Physiol.* 271 R1638–R1642 8997364 10.1152/ajpregu.1996.271.6.R1638

[B51] Monda M. Viggiano A. De Luca V. (2003). A paradoxical effect of orexin A: the hypophagia induced by hyperthermia. *Brain Res.* 961 220–228 10.1016/S0006-8993(02)03953-7 12531489

[B52] Monda M. Viggiano A. Mondola P. De Luca V. (2001). Inhibition of prostaglandin synthesis reduces hyperthermic reactions induced by hypocretin-1/orexin A. *Brain Res.* 909 68–74 10.1016/S0006-8993(01)02606-3 11478922

[B53] Monda M. Viggiano A. Viggiano A. Fuccio F. De Luca V. (2004a). Injection of orexin A into the diagonal band of Broca induces sympathetic and hyperthermic reactions. *Brain Res.* 1018 265–271 10.1016/j.brainres.2004.05.084 15276887

[B54] Monda M. Viggiano A. Viggiano A. Fuccio F. De Luca V. (2004b). Clozapine blocks sympathetic and thermogenic reactions induced by orexin A in rat. *Physiol. Res.* 53 507–513 15479129

[B55] Monda M. Viggiano A. N. Viggiano A. Viggiano E. Lanza A. De Luca V. (2005). Hyperthermic reactions induced by orexin A: role of the ventromedial hypothalamus. *Eur. J. Neurosci.* 22 1169–1175 10.1111/j.1460-9568.2005.04309.x 16176359

[B56] Morrison S. F. Madden C. J. Tupone D. (2012a). An orexinergic projection from perifornical hypothalamus to raphe pallidus increases rat brown adipose tissue thermogenesis. *Adipocyte* 1 116–120 10.4161/adip.19736 23538704 PMC3607627

[B57] Morrison S. F. Madden C. J. Tupone D. (2012b). Central control of brown adipose tissue thermogenesis. *Front. Endocrinol. (Lausanne)* 3:5 10.3389/fendo.2012.00005 PMC3292175 22389645

[B58] Morrison S. F. Madden C. J. Tupone D. (2014). Central neural regulation of brown adipose tissue thermogenesis and energy expenditure. *Cell Metab.* 19 741–756 10.1016/j.cmet.2014.02.007 24630813 PMC4016184

[B59] Muroya S. Uramura K. Sakurai T. Takigawa M. Yada T. (2001). Lowering glucose concentrations increases cytosolic Ca^2+^ in orexin neurons of the rat lateral hypothalamus. *Neurosci. Lett.* 309 165–168 10.1016/S0304-3940(01)02053-5 11514067

[B60] Must A. Spadano J. Coakley E. H. Field A. E. Colditz G. Dietz W. H. (1999). The disease burden associated with overweight and obesity. *JAMA* 282 1523–1529 10.1001/jama.282.16.1523 10546691

[B61] Nowak K. W. Mac kowiak P. Switonska M. M. Fabis M. Malendowicz L. K. (2000). Acute orexin effects on insulin secretion in the rat: in vivo and in vitro studies. *Life Sci.* 66 449–454 10.1016/S0024-3205(99)00611-6 10670833

[B62] Ogden C. L. Carroll M. D. Curtin L. R. McDowell M. A. Tabak C. J. Flegal K. M. (2006). Prevalence of overweight and obesity in the United States, 1999–2004. *JAMA* 295 1549–1555 10.1001/jama.295.13.1549 16595758

[B63] Rosin D. L. Weston M. C. Sevigny C. P. Stornetta R. L. Guyenet P. G. (2003). Hypothalamic orexin (hypocretin) neurons express vesicular glutamate transporters VGLUT1 or VGLUT2. *J. Comp. Neurol.* 465 593–603 10.1002/cne.10860 12975818

[B64] Roubenoff R. Hughes V. A. Dallal G. E. Nelson M. E. Morganti C. Kehayias J. J. (2000). The effect of gender and body composition method on the apparent decline in lean mass-adjusted resting metabolic rate with age. *J. Gerontol. A Biol. Sci. Med. Sci.* 55 M757–M760 10.1093/gerona/55.12.M757 11129399

[B65] Runge C. F. (2007). Economic consequences of the obese. *Diabetes* 56 2668–2672 10.2337/db07-0633 17601989

[B66] Sakurai T. Amemiya A. Ishii M. Matsuzaki I. Chemelli R. M. Tanaka H. (1998). Orexins and orexin receptors: a family of hypothalamic neuropeptides and G protein-coupled receptors that regulate feeding behavior. *Cell* 92 573–585 10.1016/S0092-8674(00)80949-6 9491897

[B67] Schuld A. Hebebrand J. Geller F. Pollmacher T. (2000). Increased body-mass index in patients with narcolepsy. *Lancet* 355 1274–1275 10.1016/S0140-6736(05)74704-8 10770327

[B68] Shah A. J. Su S. Veledar E. Bremner J. D. Goldstein F. C. Lampert R. (2011). Is heart rate variability related to memory performance in middle-aged men? *Psychosom. Med.* 73 475–482 10.1097/PSY.0b013e3182227d6a 21715297 PMC3307789

[B69] Shiraishi T. Oomura Y. Sasaki K. Wayner M. J. (2000). Effects of leptin and orexin-A on food intake and feeding related hypothalamic neurons. *Physiol. Behav.* 71 251–261 10.1016/S0031-9384(00)00341-3 11150556

[B70] Smith M. M. Minson C. T. (2012). Obesity and adipokines: effects on sympathetic overactivity. *J. Physiol.* 590 1787–1801 10.1113/jphysiol.2011.221036 22351630 PMC3573303

[B71] Snow A. Gozal E. Malhotra A. Tiosano D. Perlman R. Vega C. (2002). Severe hypersomnolence after pituitary/hypothalamic surgery in adolescents: clinical characteristics and potential mechanisms. *Pediatrics* 110:e74 10.1542/peds.110.6.e74 12456941

[B72] Stanley S. Pinto S. Segal J. Pérez C. A. Viale A. DeFalco J. (2010). Identification of neuronal subpopulations that project from hypothalamus to both liver and adipose tissue polysynaptically. *Proc. Natl. Acad. Sci. U.S.A.* 107 7024–7029 10.1073/pnas.1002790107 20351287 PMC2872469

[B73] Sudo M. Minokoshi Y. Shimazu T. (1991). Ventromedial hypothalamic stimulation enhances peripheral glucose uptake in anesthetized rats. *Am. J. Physiol.* 261 E298–E303 1887876 10.1152/ajpendo.1991.261.3.E298

[B74] Suzuki K. Jayasena C. N. Bloom S. R. (2012). Obesity and appetite control. *Exp. Diabetes Res.* 2012:824305 10.1155/2012/824305. PMC3415214 22899902

[B75] Sweet D. C. Levine A. S. Billington C. J. Kotz C. M. (1999). Feeding response to central orexins. *Brain Res.* 821 535–538 10.1016/S0006-8993(99)01136-1 10064843

[B76] Tentolouris N. Liatis S. Katsilambros N. (2006). Sympathetic system activity in obesity and metabolic syndrome. *Ann. N. Y. Acad. Sci.* 1083 129–152 10.1196/annals.1367.010 17148737

[B77] Thayer J. F. Lane R. D. (2007). The role of vagal function in the risk for cardiovascular disease and mortality. *Biol. Psychol.* 74 224–242 10.1016/j.biopsycho.2005.11.013 17182165

[B78] Tonhajzerova I. Javorka M. Trunkvalterova Z. Chroma O. Javorkova J. Lazarova Z. (2008). Cardio-respiratory interaction and autonomic dysfunction in obesity. *J. Physiol. Pharmacol.* 59 709–718 10.2106/JBJS.I.00705 19218698

[B79] Trivedi P. Yu H. MacNeil D. J. Van der Ploeg L. H. Guan X. M. (1998). Distribution of orexin receptor mRNA in the rat brain. *FEBS Lett.* 438 71–75 10.1016/S0014-5793(98)01266-6 9821961

[B80] Tsuneki H. Wada T. Sasaoka T. (2010). Role of orexin in the regulation of glucose homeostasis. *Acta Physiol. (Oxf.)* 198 335–348 10.1111/j.1748-1716.2009.02008.x 19489767

[B81] Van Baak M. A. (2008). Meal-induced activation of the sympathetic nervous system and its cardiovascular and thermogenic effects in man. *Physiol. Behav.* 94 178–186 10.1016/j.physbeh.2007.12.020 18281067

[B82] Van Vliet-Ostaptchouk J. V. Nuotio M. L. Slagter S. N. Doiron D. Fischer K. Foco L. (2014). The prevalence of metabolic syndrome and metabolically healthy obesity in Europe: a collaborative analysis of ten large cohort studies. *BMC Endocr. Disord.* 14:9 10.1186/1472-6823-14-9 PMC3923238 24484869

[B83] Venner A. Karnani M. M. Gonzalez J. A. Jensen L. T. Fugger L. Burdakov D. (2011). Orexin neurons as conditional glucosensors: paradoxical regulation of sugarsensing by intracellular fuels. *J. Physiol.* 589 5701–5708 10.1113/jphysiol.2011.217000 22005675 PMC3249044

[B84] Viggiano A. Viggiano A. Monda M. Turco I. Incarnato L. Vinno V. (2006). Annurca apple-rich diet restores long-term potentiation and induces behavioral modifications in aged rats. *Exp. Neurol.* 199 354–361 10.1016/j.expneurol.2006.01.001 16480716

[B85] Wang Y. Beydoun M. A. (2007). The obesity epidemic in the United States – gender, age, socioeconomic, racial/ethnic, and geographic characteristics: a systematic review and meta-regression analysis. *Epidemiol. Rev.* 29 6–28 10.1093/epirev/mxm007 17510091

[B86] Welle S. Schwartz R. G. Statt M. (1991). Reduced metabolic rate during β-adrenergic blockade in humans. *Metabolism* 40 619–622 10.1016/0026-0495(91)90053-Y 1650879

[B87] Weyer C. Snitker S. Rising R. Bogardus C. Ravussin E. (1999). Determinants of energy expenditure and fuel utilization in man: effects of body composition, age, sex, ethnicity and glucose tolerance in 916 subjects. *Int. J. Obes. Relat. Metab. Disord.* 23 715–722 10.1038/sj.ijo.0800910 10454105

[B88] Wolf G. (1998). Orexins: a newly discovered family of hypothalamic regulators of food intake. *Nutr. Rev.* 56 172–173 10.1111/j.1753-4887.1998.tb06131.x 9656726

[B89] Yach D. Suckler D. Brownel K. (2006). Epidemiologic and economic consequences of the global epidemics of obesity and diabetes. *Nat. Med.* 12 62–66 10.1038/nm0106-62 16397571

[B90] Yi C. X. Serlie M. J. Ackermans M. T. Foppen E. Buijs R. M. Sauerwein H. P. (2009). A major role for perifornical orexin neurons in the control of glucose metabolism in rats. *Diabetes* 58 1998–2005 10.2337/db09-0385 19592616 PMC2731521

[B91] Zhang S. Blache D. Vercoe P. E. Adam C. L. Blackberry M. A. Findlay P. A. (2005). Expression of orexin receptors in the brain and peripheral tissues of the male sheep. *Regul. Pept.* 124 81–87 10.1016/j.regpep.2004.07.010 15544844

[B92] Zheng H. Patterson L. M. Berthoud H. R. (2005). Orexin-A projections to the caudal medulla and orexin-induced c-Fos expression, food intake, and autonomic function. *J. Comp. Neurol.* 485 127–142 10.1002/cne.20515 15776447

